# The Effect of Professional Autonomy and Nursing Work Environment on Nurses' Patient Safety Activities: A Perspective on Magnet Hospitals

**DOI:** 10.1155/2023/5587501

**Published:** 2023-08-25

**Authors:** Songyi Yuk, Soyoung Yu

**Affiliations:** ^1^Bundang CHA Hospital, 59, Seongnam, Gyeonggi-do, Republic of Korea; ^2^College of Nursing, CHA University, 120, Pocheon-shi, Gyeonggi-do, Republic of Korea

## Abstract

**Aim:**

This study aimed to identify the effects of nurses' professional autonomy and work environment on patient safety in general hospitals. By understanding this relationship, we sought to identify practical measures to improve patient safety in the healthcare context.

**Background:**

Professional autonomy and nursing work environments have positive effects on job performance, job satisfaction, and patient outcomes.

**Methods:**

Data were collected from 200 nurses working in general hospitals, using a cross-sectional survey design. The survey was conducted in 2021 using self-report questionnaires that included the Nursing Work Environment Scale and items on professional autonomy and patient safety activities.

**Results:**

The total professional autonomy score was 162.06 (range: 60–240). The mean scores of nursing work environment and nurses' patient safety activities were 2.55 out of 4 and 4.22 out of 5, respectively. Multiple regression analysis revealed that professional autonomy (*β* = 0.234 and *p*=0.001) and nursing work environment (*β* = 0.138 and *p* < 0.05) were identified as variables that had significant effects on patient safety activities. Among the independent variables, professional autonomy had the greatest influence on patient safety.

**Conclusion:**

The findings confirm the need to improve and find ways to enhance nurses' professional autonomy and nursing work environment. *Implications for Nursing Management*. The findings confirm the need to improve and find ways to enhance nurses' professional autonomy and the nursing work environment. Based on these findings, medical institutions and nursing managers should continue to make efforts to improve the nursing work environment to enhance patient safety, especially system improvements, expand nurse autonomy within medical institutions, strengthen hospital management support to evaluate its effectiveness, and further improve government-level policies and systems.

## 1. Introduction

Patient safety is a framework for organizational activities that create an organizational culture, process, procedure, behavior, technology, and environment that consistently lowers risk, reduces the likelihood of avoidable harm and error, and, if any, reduces their impact [1]. The responsibility for patient safety management is imposed on various organizations related to patient safety; however, practical safety management activities are left to all members of the medical institution, including medical personnel. Nurses account for more than 60% of the workforce, implying an absolute effect on patient safety. In particular, many tasks involving a high frequency of accidents occur in the field of nursing. These findings suggest that nurses play key roles in patient safety management [2]. As a result of examining the factors affecting nurses' patient safety activities by dividing them into individual and organizational concepts in previous studies [2–5], attention was paid to professional autonomy and the nursing work environment.

Autonomy is associated with the ability to control one's nursing practice and is regarded as an element of professional governance [4]. Extensive empirical studies on the characteristics of magnetic working environments have been conducted [6]. Magnet recognition is rooted in a strong scientific foundation over a 20-year research and evaluation period; thus, gaining magnet recognition is the highest honor that a medical institution's nursing department can achieve [7]. The Magnet Recognition Committee of the American Nurses Credentialing Center developed magnet's recognized criteria for evaluating organizational performance from the initial 14 forces of magnetism—the factors affecting recruitment and retention—to an outcome-based model essential to a culture of excellence and innovation in nursing [8]. There are 14 forces in the forces of magnetism, including the quality of nurse leadership; even in the 2008 revised model, “autonomy” is an element of exemplary professional practice [9]. According to a scoping review published in 2021, nurses' professional autonomy can be defined as independence in decision-making and the ability to apply competencies [3]. Studies have reported that higher nurse autonomy is associated with better patient outcomes in magnet hospitals and higher nurse autonomy is linked to lower patient mortality and higher rescue success [9–11]. In addition, professional autonomy has a positive effect on nurses' job performance, including organizational commitment, job satisfaction, and work performance [12]. Various factors, including better working conditions and intangible compensation, can help address nurses' manpower shortages, which have persisted since and after the coronavirus disease 2020 pandemic, and professional autonomy is known to belong to this intervention [13].

Another important component of the Magnet® model is the nursing work environment, which positively affects the organizational culture and patient outcomes [5]. The term “magnet hospital” was officially coined and defined by the American Nurses Credentialing Center to describe hospitals that meet certain criteria indicating that they have a “magnetic work environment” for nurses [9]; in other words, the nursing work environment is a crucial factor in magnet recognition. Identifying the aspects of the nursing work environment that affect nurses' turnover intentions is important because they can be useful in promoting nursing practices and policies [12]. Studies related to nursing work environments have shown that better work environments contribute to lower mortality, better patient outcomes [9], and higher scores of Hospital Consumer Assessment of Health Providers and Systems (HCAHPS). In particular, it is necessary to pay attention to research results that analyze the relationship between the nursing work environment and attitudes toward patient safety and error reporting. Nurses' perceptions of the working environment and patient-safety culture were positively correlated with their attitudes toward accident reporting. In addition, it was reported that improving nurses' attitudes toward accident reporting could be achieved through a wide range of approaches, including improving the working environment and patient-safety culture [14]. Another study suggested the possibility of improving the patient-safety culture by managing the work environment [15]. Similar results have been reported in other studies related to the nursing work environment and patient-safety culture [16, 17].

Based on research results on professional autonomy and the nursing work environment [2–5], it is necessary to evaluate how professional autonomy and the nursing work environment affect nurses' patient safety activities. The findings of this study can provide a basis for improving nurses' professional autonomy and work environments. Therefore, this study aimed to identify the effects of nurses' professional autonomy and work environment on patient safety activities in general hospitals. By understanding this relationship, we sought to identify practical measures to improve patient safety in the healthcare context.

## 2. Materials and Methods

### 2.1. Purpose and Study Design

This cross-sectional study aimed to investigate the professional autonomy and work environment of nurses working in the acute care sector and to identify their effects on patient safety activities. A cross-sectional survey was conducted among nurses working in general hospitals in Korea.

### 2.2. Study Setting and Sample Size

This study collected data from nurses with more than one year of experience working at three general hospitals in Seoul and the metropolitan area, one general hospital with approximately 900 beds, and one hospital located in Gyeonggi-do. The G^*∗*^Power 3.1.9.7 program was used to calculate the sample size required for this study. The number of participants for multiple regression analysis was 172, based on an effect size (*f*^2^) 0.15, power 95% confidence in two-tailed tests, and 10 predictors. Thus, 200 nurses were surveyed, considering the dropout rate of 10%.

### 2.3. Measures

#### 2.3.1. Professional Autonomy

The Schutzenhofer Professional Autonomy Scale (SPAS) developed by Schutzenhofer [18] and translated by Han et al. [19] was used. It consists of 30 items, answered on a 4-point Likert scale, with higher scores indicating higher professional autonomy. Each item is weighted from 1 to 3 according to the level of autonomy. The level of professional autonomy is indicated in 3 stages, with each stage comprising 10 questions. Low-level autonomy (10 items) weighted 1, middle-level autonomy (10 items) weighted 2, and high-level autonomy (10 items) weighted 3. The total scores ranged from 60 to 240 points, with 60–120 points and 181–240 points indicating low and high levels, respectively. In Schutzenhofer's study [18], the reliability of the tool at the time of development was 0.92, and in Han et al.'s study [19], it was 0.91. In the present study, it was 0.87.

#### 2.3.2. Practice Environment Scale of the Nursing Work Index (PES-NWI)

The Practice Environment Scale of the Nursing Work Index (PES-NWI) developed by Lake [20] was adapted to suit the situation in Korea by Cho et al. [21], and the Korean version of the Nursing Work Environment Measurement Tool (K-PES-NWI) was used. It comprises 29 questions, and each question is answered on a 4-point Likert scale; the higher the score, the more positively the nurse recognizes their work environment. Cronbach's *α* of the five subscales of the PES-NWI is as follows: “nurses' participation in hospital affairs (nine questions)” was 0.83, “nursing foundations for quality of care (nine questions)” was 0.80, “nurse manager ability, leadership, and support for nurses (four questions)” was 0.84, “staffing and resource adequacy (four questions)” was 0.80, and “collegial nurse-physician relations (three questions)” was 0.71 [20]. In the study by Cho et al. [21], the reliability of the tool at the time of development was 0.93, and Cronbach's *α* in this study was 0.92.

#### 2.3.3. Patient-Safety Activities

The items for patient safety activities were developed by Lee [22]. There are a total of 40 items across eight subscales, including patient identification, surgery and procedure, safe environment, and infection. Each question is measured on a Likert scale of 1 point (not at all) and 5 points (very much), and the higher the score, the higher the degree of patient safety activities of the nurse. At the time of development of the tool, Cronbach's *α* was 0.92 [22], and in this study, it was 0.96; in this study, Cronbach's *α* for each of the eight subareas was identified as follows: “patient identification” was 0.80, “verbal orders” was 0.77, “medication administration” was 0.75, “procedure and surgery” was 0.90, “safe environment” was 0.78, “infection control” was 0.86, “fall prevention” was 0.87, “pressure ulcer prevention” was 0.88, and “emergency preparedness” was 0.91.

### 2.4. Data Collection

Two hundred nurses working at three general hospitals were recruited using the convenience sampling technique, and they were administered a questionnaire. Of these, 194 questionnaires were used for the final data analysis, after excluding incomplete questionnaires. First, after obtaining permission from the Research Ethics Review Committee of the hospital, the research plan, questionnaire, an explanation of the study, ethics approval notice, and request for collection of research data were submitted to the hospital, and the purpose of this study was explained to the nursing department head. The study participants were provided with an explanation and a written consent form, which stated the study's purpose and instructions on how to respond to the questionnaire. The survey response took about 10–15 minutes. Only those who voluntarily agreed to participate in the study and who provided written consent were included in the survey. Forwarding envelopes were used to ensure anonymity, and data were collected between September 15 and October 29, 2021.

### 2.5. Data Analysis

Data were analyzed using SPSS 25.0 (IBM Corp., Armonk, NY, USA). Descriptive statistics were used to determine patients' general characteristics and scores on the study measures. To test the relationship between professional autonomy, nursing work environment, and patient safety activities according to participants' general characteristics, a *t*-test and analysis of variance were performed, and Scheffé's test was performed as a post hoc test. Pearson's correlation coefficients were used to determine the correlation between professional autonomy, nursing work environment, and patient safety activities, as perceived by the participants. The Durbin-–Watson statistic was 1.912; thus, there was no autocorrelation problem in the autocorrelation verification of the errors. The variance expansion factor (VIF) between the independent variables was confirmed to be less than 10 at 1.009–1.060, indicating no multicollinearity. Therefore, the research data were suitable for regression analysis. Multiple regression analysis was performed on the factors affecting the participants' patient safety activities.

### 2.6. Ethical Considerations

This study collected data after obtaining approval from the CHA Hospital Medical Research Ethics Review Committee (IRB no. CHAMC 2021-06-042-002). The consent form was signed only by those who fully understood and voluntarily agreed to participate in the study, after being explained the study purpose and procedure, participants' rights and autonomy regarding participation, the confidentiality of the responses, and absence of any disadvantages for not participating or withdrawing from the study. When entering the data, a unique number was assigned and coded to protect the participants' personal information, and research-related records were kept and destroyed in accordance with the Enforcement Rules of the Bioethics Act and the Enforcement Decree of the Personal Information Protection Act.

## 3. Results

### 3.1. General Characteristics of the Participants

As shown in Table 1, the majority were women, 20–29 years old, with a bachelor's degree, nonreligious, and unmarried. Regarding workplace positions, the number of charge nurses was higher than that of staff nurses. The departments varied and included general wards, integrated nursing care service wards, and intensive care units. In terms of total clinical experience, 3–6 years of experience was most common. In terms of current department experience, 1–3 years was most common. Regarding the type of work, the majority was performing shift duties (Table 1).

### 3.2. Nurses' Professional Autonomy, Nursing Work Environment, and Patient Safety Activities of Participants

As shown in Table 2, the total score for nurses' professional autonomy was 162.06 which was middle level. The total score ranges from 60 to 240 points, with 60–120 points, 121–180 points, and 181–240 points indicating low, middle, and high levels, respectively. The mean score for the nursing work environment was 2.55 out of 4, while the mean score for patient safety activities was 4.22 out of 5. In terms of the nursing work environment, based on Lake and Friese's [23] finding that a value of 2.5 or more can be viewed as a theoretical intermediate value, the result of this study, 2.55 points, belongs to “favorable.” Table 3 presents the differences in nurses' professional autonomy, nursing work environment, and patient safety activities according to their general characteristics. Regarding professional autonomy, significant differences were found in terms of education and work type. The nursing work environment differed significantly according to position, department, clinical experience, and current department experience. Patient safety activities differed significantly according to marital status (Table 3).

### 3.3. Relationship between Nurses' Professional Autonomy, Work Environment, and Patient Safety Activities

Professional autonomy showed significant positive correlations with the nursing work environment and patient safety activities. In addition, the nursing work environment showed a significant positive correlation with patient safety activities (Table 4).

### 3.4. Factors Influencing Patient Safety Activities

As shown in Table 5, in this study, multiple regression analysis was conducted to determine the factors affecting participants' patient safety activities. Professional autonomy, nursing work environment, marital status, and patient safety activities were included in the regression model. Multiple regression analysis revealed that marital status (*β* = 0.191 and *p* < 0.001), professional autonomy (*β* = 0.234 and *p*=0.001), and nursing work environment (*β* = 0.138 and *p* < 0.05) were subsequently identified as variables that had statistically significant effects on patient safety activities and explained about 11.5% of the variance in patient safety activities. Among the independent variables, professional autonomy was found to have the greatest influence on patient safety activities.

## 4. Discussion

This study was conducted to identify professional autonomy and nursing working environment, which are major criteria in the forces of magnetism, and to find a basis for improving nurses' working environment and patient safety activities by confirming their effects on hospital nurses' safety activities.

### 4.1. Professional Autonomy and Patient Safety Activities

In this study, nurses' professional autonomy score was 162.06 points, which was relatively lower than that reported in previous studies [24–26] that used the same tool. While this study measured the overall professional autonomy of general ward nurses, it is likely because professional nurses from previous studies (i.e., oncology nurses) conducted patient care based on more professional expertise and skills. In addition, nurses working shifts had high professional autonomy scores. Nurses in wards, intensive care units, and emergency centers take care of patients with a wide range of severity and treatment and perform relatively more continuous and direct nursing through shift work; therefore, the level of professional autonomy was measured in this study. This study confirmed that professional autonomy was the most influential factor in patient safety activities. In other words, measures should be taken to increase patient safety activities through step-by-step autonomy-improvement training and system improvements. In this study, there was no significant difference in professional autonomy according to general characteristics, other than work type. Cornock [27] reported that professional autonomy is affected by the limited responsibilities of nurses, the power of other professions, and lack of confidence in knowledge; based on the results of this study, it was expected that the hospital's organizational culture and education system would be more influenced than general characteristics such as age, religion, and marital status. Therefore, organizational support is needed to encourage nurses to exercise a high level of autonomy through step-by-step autonomy-improvement training [25, 28] and to analyze the characteristics of nurses with a high level of autonomy.

### 4.2. Nursing Working Environment and Patient Safety Activities

The average level of the nursing work environment in this study was an average of 2.55 points out of 4. Lake and Friese [23] considered that if the score was 2.5 or higher, the nurse agreed that the work environment was good for working. However, it is a low score compared to 2.65 points for nonmagnet hospitals in the United States, and it is a result that shows a greater difference from 2.95 points for magnet hospitals [20]. It was found that the score was similar to that of a study [29, 30] that measured the nursing work environment of nurses in general hospitals in Korea using the same tool. These results confirm that various efforts are needed to improve the nursing work environment. Among the subitems of the nursing work environment, the score in the “staffing and resource adequacy” area was the lowest at 2.09, similar to the results of studies using the same tool [29–31]. Questions in this area include “Adequate support services allow me to spend time with my patients,” “Enough registered nurses to provide quality patient care,” and “Enough time and opportunity to discuss patient care problems with other nurses.” It is widely accepted that appropriate nursing personnel are associated with high-quality patient care, including patient safety, in conjunction with a healthy working environment [32]. In addition, regarding the nursing work environment, which is a major variable in this study, some evidence suggests that the RN staffing level is part of a causal chain linking magnet states to quality improvement [23, 32]. Despite this evidence, in South Korea, the number of patients in charge of one nurse is more than 1 : 10. The nursing work environment related to patient safety is very poor, and the reality of this work environment is reflected in this study. These results should be improved because there are no magnetic hospitals in South Korea. Several countries, including Korea, are striving to improve the working environment of nurses and improve welfare; however, it can be confirmed that the shortage of nurses is still a serious problem. The issue of nursing staff is not only a high turnover problem but also an improvement in the overall treatment, such as poor working conditions and salaries, as well as improving nurses' job satisfaction and quality of life, which can affect people's health. Therefore, practical institutional improvement should be made.

### 4.3. Patient Safety Activities

The average level of patient safety activities in this study was an average of 4.42 points out of 5. This is a higher score than other studies [33–35], which is considered a positive result of strengthening awareness of patient safety management activities through the placement of patient safety personnel and periodic medical institution accreditation systems. Although we should be careful in interpreting the findings, it can be interpreted as hospitals researched in this study continue to receive hospital accreditation, and various indicators emphasizing patient safety are included as the main items of hospital accreditation in Korea. However, considering that the evaluation tool was conducted in a self-report manner, it is necessary to consider the possibility that participants answered more positively than the current nursing activities [33], and future repeated studies will be needed. The level of each subarea of patient safety activities was found to be high in the order of falls (4.70), infections (4.67), and surgery and procedures (4.63). These results are similar to those of previous studies [33–35]. This is considered to reflect the results of continuous fall prevention management through fall assessment tools, as not only medical institution accreditation but also the report rate of fall occurrence is one of the main assessment indicators for patient safety. However, the lowest level was the safe environment (3.98), and in previous studies, the safe environment items were measured at a low level [33, 34]. Since the hospital environment can be directly related to the safety of patients in the case of a disaster or emergency, regular inspections, practical environmental improvement, and systematic safety education should be strengthened to mitigate these risks.

We found a significant positive correlation between professional autonomy, the nursing work environment, and nurses' patient safety activities. This finding is consistent with the results of previous studies on the effect of nurses' professional autonomy on patient safety management activities [3, 23, 36, 37]. Therefore, raising nurses' levels of professional autonomy and improving the nursing work environment can enhance the quality of nursing care by increasing the performance rate of patient safety activities. These two factors have already been proven by many magnet hospitals [6, 7, 9, 10, 12], and from the point of view of the magnet hospital, it is an essential element for hospitals, and it is now an essential element for improving patient safety.

### 4.4. Limitations

First, this study involved a survey of nurses in only three general hospitals, and the study collected and analyzed data, so there is a limit in generalizing the results. Second, because a self-report questionnaire was used, it should be interpreted considering the limitations of the self-report questionnaire method, which can be different from the actual one, and there may be limitations owing to the convenience sampling of the study participants. The control of respondents' biases was supplemented by calculating the number of samples required for the survey study using the G^*∗*^Power program, and it was possible to collect data only from nurses who wanted to participate in the survey after notifying all nurses in the three hospitals; so there was a limit to random selection. Finally, the values presented in the results of this study are statistically significant, but they are not high or large; therefore, it is necessary to check them through future studies.

## 5. Conclusions

This study was conducted to confirm the effect of nurses' professional autonomy and nursing work environment on nurses' patient safety activities. The results showed that professional autonomy and nursing work environment were identified as variables that had a significant relationship with patient safety activities. The findings confirm the need to improve and find ways to enhance nurses' professional autonomy and nursing work environment. This study is different from previous ones in which it allows nursing managers and hospital administrators to consider practical measures to improve patient safety in the current healthcare setting where patient safety is emphasized.

### 5.1. Implications for Nursing Management

Improving professional autonomy improves the ability to maintain decision-making independence, applying one's capabilities within the scope of practice and nursing work environments. Consequently, another important component of the Magnet® model is associated with nurses' patient safety activities. Based on these findings, medical institutions and nursing managers should continue to make efforts to improve the nursing work environment and patient safety, prepare various systems to expand nurse autonomy within medical institutions, strengthen hospital management support to evaluate its effectiveness, and further improve government-level policies and systems.

## Figures and Tables

**Table 1:** General characteristics of the participants.

Characteristics	Category	*n*	%
Gender	Male	11	5.67
	Female	183	94.33

Age (years)	20–29	118	60.82
	30–39	66	34.02
	40–49	8	4.12
	≥50	2	1.03

Education	Associate bachelor	11	5.67
	Bachelor	172	88.66
	Master	11	5.67

Religion	Yes	66	34.02
	No	128	65.98

Marital status	Married	43	22.16
	Single	148	76.29
	Others	3	1.55

Position	Staff nurse	81	41.75
	Charge nurse	113	58.25

Department	General ward	14	7.22
	Integrated nursing care service ward	59	30.41
	ICU	27	13.92
	OR	23	11.86
	RR	22	11.34
	EMC	26	13.40
	Outpatient clinic	18	9.29
	Others	5	2.58

Clinical experience (years)	1-<3	58	29.90
	3-<6	60	30.93
	6-<10	39	20.10
	10≤	37	19.07

Current department experience	1-<3	81	41.75
	3-<6	63	32.47
	6-<10	34	17.53
	10≤	16	8.25

Type of work	Shift duty	173	89.18
	Daytime only	21	10.82

*N* = 194.

**Table 2:** Descriptive statistics of professional autonomy, nursing work environment, and nurses' patient safety activities.

Variables	Items	M ± SD	Total ± SD	Min	Max	Range
Professional autonomy	30		162.06 ± 17.76	102	225	60∼240
Low level	10	2.71 ± 0.37		1.20	3.70	1∼4
Middle level	10	5.39 ± 0.69		3.80	8.00	2∼8
High level	10	8.11 ± 0.95		4.20	10.80	3∼12
Nursing work environment (K-PES-NW)	29	2.55 ± 0.40		1.38	3.66	1∼4
Nurse participation in hospital affairs	9	2.40 ± 0.48		1.22	4.00	
Nursing foundations for quality of care	9	2.75 ± 0.40		1.67	3.67	
Nurse manager ability, leadership, and support	4	2.80 ± 0.51		1.00	4.00	
Staffing and resource adequacy	4	2.09 ± 0.59		1.00	4.00	
Collegial nurse-physician relations	3	2.69 ± 0.57		1.00	4.00	
Nurses' patient safety activities	40	4.42 ± 0.42		2.15	5.00	1∼5
Patient identification	7	4.48 ± 0.41		2.71	5.00	
Verbal orders	3	4.50 ± 0.59		2.33	5.00	
Medication administration	7	4.29 ± 0.51		2.57	5.00	
Procedure and surgery	4	4.63 ± 0.52		2.00	5.00	
Safety environment	3	3.98 ± 0.83		1.67	5.00	
Infection control	3	4.67 ± 0.52		1.00	5.00	
Fall prevention	3	4.70 ± 0.50		1.67	5.00	
Pressure ulcer prevention	3	4.49 ± 0.64		1.33	5.00	
Emergency preparedness	7	4.29 ± 0.66		1.29	5.00	

*N* = 194.

**Table 3:** Differences in professional autonomy, nursing work environment, and nurses' patient safety activities.

	Characteristics	*n*	Professional autonomy		Nursing work environment		Nurses' patient safety activities	
			M ± SD	*t* or *F* (*p*) Scheffé	M ± SD	*t* or *F* (*p*) Scheffé	M ± SD	*t* or *F* (*p*) Scheffé
Gender	Male	11	168.27 ± 25.49	1.196 (0.233)	2.69 ± 0.56	1.145 (0.254)	4.34 ± 0.44	−0.634 (0.527)
	Female	183	161.68 ± 17.22		2.54 ± 0.39		4.42 ± 0.42	

Age (years)	20–29	118	162.12 ± 17.80	0.926 (0.429)	2.59 ± 0.41	1.379 (0.251)	4.45 ± 0.37	0.646 (0.586)
	30–39	66	161.45 ± 17.93		2.48 ± 0.40		4.38 ± 0.51	
	40–49	8	169.63 ± 17.21		2.45 ± 0.28		4.31 ± 0.40	
	≥50	2	148.00 ± 2.82		2.71 ± 0.02		4.48 ± 0.28	

Education	Associate bachelor	11	165.00 ± 16.19	3.162 (0.045)^n/a^	2.63 ± 0.33	0.251 (0.778)	4.34 ± 0.32	1.602 (0.204)
	Bachelor	172	161.08 ± 17.19		2.55 ± 0.41		4.44 ± 0.41	
	Master	11	174.45 ± 24.10		2.51 ± 0.44		4.23 ± 0.63	

Religion	Yes	66	161.38 ± 16.87	−0.381 (0.704)	2.56 ± 0.36	0.289 (0.773)	4.42 ± 0.46	−0.052 (0.958)
	No	128	162.41 ± 18.26		2.55 ± 0.42		4.42 ± 0.40	

Marital status	Married^a^	43	161.09 ± 19.25	1.126 (0.326)	2.46 ± 0.40	2.190 (0.115)	4.27 ± 0.55	4.137 (0.017)^*∗*^^a<b^
	Single^b^	148	162.03 ± 17.36		2.57 ± 0.40		4.47 ± 0.37	
	Others^c^	3	177.00 ± 13.00		2.85 ± 0.22		4.22 ± 0.23	

Position	Staff nurse	81	161.40 ± 18.00	−0.438 (0.662)	2.64 ± 0.40	2.532 (0.012)^*∗*^	4.43 ± 0.36	0.169 (0.866)
	Charge nurse	113	162.53 ± 17.66		2.49 ± 0.40		4.42 ± 0.46	

Department	General ward	14	161.57 ± 13.98	1.306 (0.249)	2.33 ± 0.37	2.578 (0.015)^*∗*^^n/a^	4.25 ± 0.50	1.133 (0.344)
	Integrated nursing care service ward	59	166.39 ± 14.42		2.56 ± 0.40		4.53 ± 0.31	
	ICU	27	164.89 ± 18.75		2.57 ± 0.40		4.45 ± 0.42	
	OR	23	158.22 ± 21.48		2.53 ± 0.50		4.32 ± 0.41	
	RR	22	159.14 ± 21.81		2.82 ± 0.36		4.38 ± 0.44	
	EMC	26	160.77 ± 19.62		2.43 ± 0.33		4.38 ± 0.64	
	Outpatient clinic	18	154.83 ± 13.95		2.53 ± 0.36		4.43 ± 0.28	
	Others	5	160.20 ± 17.57		2.61 ± 0.23		4.44 ± 0.36	

Clinical experience (years)	1-<3	58	159.98 ± 16.77	0.932 (0.426)	2.66 ± 0.39	2.984 (0.033)^*∗*^^n/a^	4.43 ± 0.36	1.438 (0.233)
	3-<6	60	163.60 ± 18.84		2.56 ± 0.38		4.46 ± 0.39	
	6-<10	39	164.87 ± 14.17		2.44 ± 0.38		4.48 ± 0.44	
	10≤	37	159.84 ± 20.70		2.49 ± 0.45		4.30 ± 0.53	

Current department experience	1-<3^a^	81	161.17 ± 17.84	0.421 (0.738)	2.66 ± 0.39	5.607 (0.001)^*∗∗*^^a>c^	4.44 ± 0.37	1.059 (0.368)
	3-<6^b^	63	163.49 ± 18.24		2.55 ± 0.37		4.46 ± 0.37	
	6-<10^c^	34	163.06 ± 15.73		2.35 ± 0.35		4.40 ± 0.37	
	10≤^d^	16	158.75 ± 20.39		2.46 ± 0.52		4.25 ± 0.69	

Type of work	Shift duty	173	163.02 ± 18.01	2.183 (0.030)^*∗*^	2.55 ± 0.41	0.061 (0.952)	4.42 ± 0.44	−0.366 (0.716)
	Daytime only	21	154.14 ± 13.54		2.55 ± 0.33		4.45 ± 0.26	

^*∗*^ *p* < 0.05,  ^*∗∗*^*p* < 0.01, and ^*∗∗∗*^*p* < 0.001. *N* = 194.

**Table 4:** Correlations among professional autonomy, nursing work environment, and nurses' patient safety activities.

Variables	Professional autonomy	Nursing work environment	Nurses' patient safety activities
	*r* (*p*)	*r* (*p*)	*r* (*p*)
Professional autonomy	1		
Nursing work environment	0.220 (0.002)^*∗*^	1	
Nurses' patient safety activities	0.268 (≤0.01)^*∗∗*^	0.208 (0.004)^*∗*^	1

^*∗*^ *p* < 0.01,  ^*∗∗*^*p* < 0.001. *N* = 194.

**Table 5:** Multiple linear regression for nurses' patient safety activities.

Variables	*B*	SE	*β*	*t*	*p*	VIF
(Constant)	3.012	0.294		10.260	≤0.01	
Marital status^*∗*^	0.189	0.067	0.191	2.804	0.006	1.009
Professional autonomy	0.006	0.002	0.234	3.375	0.001	1.051
Nursing work environment	0.144	0.073	0.138	1.977	0.049	1.060

*F* value	9.384					
*p*	<0.001					
*R* ^2^	0.129					
Adj. *R*^2^	0.115					
Durbin–Watson	1.912					

^*∗*^Reference group: marital status = married. *N* = 194.

## Data Availability

The datasets generated and/or analyzed during the current study are not publicly available due to institutional restrictions but are available from the corresponding author upon request.
